# Supplementary material for: Phenotypes to remember: Evolutionary developmental memory capacity and robustness
Source: PLoS Comput Biol. 2020 Nov 30;16(11):e1008425. doi: 10.1371/journal.pcbi.1008425 (PMC7703877; doi:10.1371/journal.pcbi.1008425)
Supplement: Attachment — Submitted filename: renamed_6c9c8.pdf [file pcbi.1008425.s002.pdf]

Review of the manuscript “Phenotypes to remember: Evolutionary developmental memory capacity and robustness”, by Szilágyi et al.

In the manuscript “Phenotypes to remember: Evolutionary developmental memory capacity and robustness”, the authors considered a gene-regulation network model that maps the initial patterns to given output patterns, in analogy with the neural network models, and then studied how evolution shapes such network.

I appreciate the authors’ motivation and relevance of the results to cell and developmental biology. Still, there are some issues to be revised, before the manuscript is considered as publication.

(1)The Introduction presents a general grand view to connect the two celebrated studied by Turing. I understand the motivation well, but as compared with this grand view, but there exist extensive publication discussing such direction to bridge the gene-expression dynamical systems and neural-network type model, as both include a threshold-type dynamics. None of them are cited unfortunately, though. For instance,

Kauffman, S.A. J. Theor. Biol. 22 (1969) 437

Glass L. and Kauffman S. A., J. Theor. Biol., 39 (1973) 103.

Mjolsness E., Sharp D. H. and Reisnitz J., J. Theor. Biol., 152 (1991) 429.

Salazar-Ciudad I., Newman S. A. and Sole R. V., Evol. Dev., 3 (2001) 84.

Ciliberti S., Martin O.C., and Wagner A. (2007)PLOS Computational Biology, {¥bf 3}, e15.

Kaneko K., PLoS ONE, 2 (2007) e434.

Indeed, there are related books by the above authors (Kauffman, Newman, Wagner, Kaneko), that include general discussion on dynamical-systems approach to development.

(2)Although, the authors discuss connection with, and possible difference from, neural networks (NN), somehow the related studies on NN are not mentioned.

*The first term in the matrix M in eq (3) or (4) is nothing but the Hopfield connection, ¥xi¥xi (by suitably transforming [-1,1]to [0,1]; e.g., by setting ¥sigma=1/2 and setting ¥xi=a-1/2), whereas Hopfieild’s paper (Hopfield, J. J. (1982)PNAS 79, 2554-2558) is not cited, even in the Table. Of course, the connection of Hopfield model is symmetric, but there are several papers that extend the connection to make it asymmetric. (Indeed, the originally NN studies take the asymmetric connection).

* Indeed the asymmetric coupling form here is quite similar with that adopted by the study by Kurikawa T. and Kaneko K. (Europhysics Letters, 98(2012), 48002; PLoS Comp Biol.

9(2013) e1002943) , where NN in which the output pattern ¥xi is attracted under the input pattern ¥eta is designed (or learned) by setting the matrix (¥xi+¥eta)(¥xi-¥eta).　Note that the matrix eq.4 by setting e-1/2=¥eta a-1/2=¥xi has the form (¥xi+¥eta)¥xi.　Hence, the difference between eq.(4) and Kurikawa's model is just the absence of -¥eta term. Indeed, in the latter model, the input ¥eta continues to be applied, whereas in the authors' study it is applied only as an input, which leads to the absence of -¥eta term.

*On the other hand, if the connection is sparse, (or if there is no overlap in the input and output), the model could be considered as an extension of the Perceptron or hetero-associative memory model, where the connection term {¥bf a}{¥bf e} is generally adopted.

Indeed, in the example the authors showed in the manuscript, there are no common components between e and a. I wonder if the model includes this non-overlapping as a constraint, or not.

(3)The memory capacity in NN has been extensively studied, whereas the capacity in the present model is not mentioned.　As far as I guess from the result of N=50,100, 250, in Fig.3, the capacity in the example is about ~0.04N. It will be relevant to mention the capacity, as well as its dependence on the parameters, say sparsity ¥sigma.

(4)It is interesting that the evolution shapes the connection matrix similar to the analytically designed one. Still, it should be noted that the evolution of GRN has been investigated extensively (say, the last three papers in (1)), as well as in recent publications to evolve an input-output relationship in GRN.

(5) Although the authors mention the similarity between the evolved network and analytically designed one in Fig.4, the results in Fig.6 show clear difference between the two. Of course, as the two networks are different, the behavior could be different. Still, it is recommended to discuss possible origin of the discrepancy. For instance, the evolved network can include some additional connections that have neutral influence.　If that is the origin of discrepancy, a possible procedure would be to extract the core part from the evolved network (i.e., remove paths, as long as the fitness is not decreased, as adopted in some earlier studies), and compare the behavior of the core network with that by the analytically designed one.

(6) Minor: In the caption of Fig.4, the authors refer to eq.(3).　I wonder whether the analytic estimated is based on eq(4).　Also some symbols K, ¥mu are not defined, if I have not looked over (K is a typos for N?).
