# Supplementary material for: Phenotypes to remember: Evolutionary developmental memory capacity and robustness
Source: PLoS Comput Biol. 2020 Nov 30;16(11):e1008425. doi: 10.1371/journal.pcbi.1008425 (PMC7703877; doi:10.1371/journal.pcbi.1008425)
Supplement: Attachment — Submitted filename: Answers.docx [file pcbi.1008425.s003.docx]

Editor:

Dear Professor Szathmáry,

Dear Professor Newman,

Herewith we are submitting our revisions and comments to the Reviewers. We respond also to you letter in the same style – see our reflections between your paragraphs below.

Thank you very much for submitting your manuscript "Phenotypes to remember: Evolutionary developmental memory capacity and robustness" for consideration at PLOS Computational Biology.

As with all papers reviewed by the journal, your manuscript was reviewed by members of the editorial board and by several independent reviewers. In light of the reviews (below this email), we would like to invite the resubmission of a significantly-revised version that takes into account the reviewers' comments.

Please note that apart from Reviewer #1's narrative comments below he also provided an annotated copy of your manuscript (see attachment).

We have considered the annotated copy also.

Reviewer #2 asks you to cite earlier work by Kauffman, Kaneko, and Newman. I would elaborate on this by noting that Kauffman and Kaneko have each presented gene regulatory network systems, which like the one in the present manuscript, generates systems of hierarchically nested attractors that serve as models of development. Newman, in contrast, has presented not a dynamical network treatment, but a perspective in which inherent physical properties of developmental systems provide a basis for understanding recurrent features in evolution. This is also a precedent for the present work.

Indeed. We have inserted the necessary citations,

Newman has also recently presented a critical view of the sufficiency of dynamical systems models for evolutionary simulations (doi: 10.1016/j.jtbi.2019.110031). The authors may or may not wish to address this critique.

Exciting point – yet we felt we should not overstretch the scope of the present paper. But we shall return to similar issues in the near future.

We cannot make any decision about publication until we have seen the revised manuscript and your response to the reviewers' comments. Your revised manuscript is also likely to be sent to reviewers for further evaluation.

When you are ready to resubmit, please upload the following:

[1] A letter containing a detailed list of your responses to the review comments and a description of the changes you have made in the manuscript. Please note while forming your response, if your article is accepted, you may have the opportunity to make the peer review history publicly available. The record will include editor decision letters (with reviews) and your responses to reviewer comments. If eligible, we will contact you to opt in or out.

[2] Two versions of the revised manuscript: one with either highlights or tracked changes denoting where the text has been changed; the other a clean version (uploaded as the manuscript file).

Important additional instructions are given below your reviewer comments.

Please prepare and submit your revised manuscript within 60 days. If you anticipate any delay, please let us know the expected resubmission date by replying to this email. Please note that revised manuscripts received after the 60-day due date may require evaluation and peer review similar to newly submitted manuscripts.

Thank you again for your submission. We hope that our editorial process has been constructive so far, and we welcome your feedback at any time. Please don't hesitate to contact us if you have any questions or comments.

Thanks a lot for kindly and considerately acting as Guest Editor for our paper.

Kind regards,

Eörs Szathmáry et al.

Sincerely,

Stuart A Newman

Guest Editor

PLOS Computational Biology

Reviewer #1: This is a very clear and well-developed paper. It develops work on the functional equivalence of learning and evolution - specifically, in the context of GRNs and correlation learning, and the ability to evolve GRNs that store multiple phenotypic memories. The work is carried out carefully and presented clearly - I see no technical problems. The results are clear and not overstated. The finding - that the functional equivalence of evolved GRNs and correlation learning models can be deepened and extended - is significant. Previous work in this area used simplifying assumptions, and assessing whether the effects depend on these assumptions is important. The work also extends the scope of evolved behaviours from autoassociative to heteroassociative - and this is a significant extension. The following comments are therefore minor/suggestions for improvements.

We are very grateful for the in-depth reading of our manuscript. We really appreciate your thought-provoking notes, comments and suggestions. Please find our answers and comments below. We took into account almost all of your suggestions in the pdf annotations.

I like an introduction to be clear about the aims and claims. As it is, the intro is excellent at setting the scene but it could be more clear about the hypotheses that will be tested or the questions that will be answered. This has a knock-on effect later in the paper.

We extended the Introduction by a paragraph that summarizes our aims and goals. We hope that will help the reader to put our results into context.

It seems like the aim is primarily to figure out whether/how the simplifying assumptions of prior work, compared to a more realistic modelling set-up for the GRN, matters to the idea of evolution learning multiple developmental memories. It turns out, by the end of the paper, that the analogy remains sound, but there are some interesting differences along the way. The results also show that the task can be extended to heteroassociative tasks which hasn’t been done before. Clarity on the balance of these two findings could improve the paper. Specifically, if the aim of the paper was really to detail the impact of these different implementational assumptions, it would have been valuable to compare the two models on the same task. I don’t think that the non-negative state models work on the tasks of earlier work. And you’ve showed that the non-negative state models do work on your task. But we don’t have a side-by-side comparison of both models on both tasks. Likewise, you suggest that the non-negative state model results in more neutrality and that this increases robustness (compared to the model with signed states). I don’t think its clear that the signed state model has a problem with robustness despite having no neutrals. And we don’t have a side-by-side comparison. However, if the claim of the paper is that these differences don’t matter much – ie that the analogy is basically sound, and the task space can be extended, then a side-by-side comparison is not needed. I think that the implementational details do matter to some tasks – and your paper is very useful in helping us think that through more carefully. And the implementational details sometimes don’t matter - and you’ve shown a case where they don’t. So, both points are worth making. Yet, I think the balance in the writing at present is a little bit towards the former, whereas the results better support the latter. So, perhaps the paper could be improved if you stated your aims/claims/questions/hypotheses more clearly at the beginning in the intro.

We made modifications in the introduction to highlight that the main goal of our paper is to demonstrate the evolutionary emergence of developmental pathways in the context of GRNs, which involves both the auto- and heteroassociative tasks (see previous point). Therefore we don’t want to make a side-by-side comparison between the two model contexts. We noted that the presence of neutral elements may add an “intrinsic” surplus robustness only if the state vectors are very sparse. In this case there are some matrix elements “opposite to” zero expression vector elements (those *m*_{*ij*} elements where *e*_*j*=0 *and* *a*_*j*=0 in all environments). The quantification and comparison of this effect with other models is not the aim of our paper. Such a quantitative comparison can be an interesting question for further research.

In line with your suggestion we made modifications to shift the emphasis in the text accordingly. We removed the relevant line of Table 1. We also removed the sentence “*This robustness is attributable to the high number of neutral elements (correspond to the zero values in state vectors) of the interaction matrix.*” from the Results section as it is valid only if the system is very sparse. Moreover we extended the summarizing sentence in the Discussion as follows: “*This feature turns out to increase the robustness of the system against the disturbance of interaction coefficients only if the system is very sparse, that guarantees the commonly zero elements in embryo and adult states.*”

A technical point relating to contrast of signed and unsigned models. You say (Table 1) that signed states will result in Hebbian learning and symmetric interaction terms (as observed in prior work). But the evolved interactions in that work are symmetric because there is no reason for them to be asymmetric – not because they have to be symmetric or because Hebb’s rule is applied. Hebb’s rule isn’t applied, of course – and they aren’t forced to be symmetric - but when there is no reason to be asymmetric, symmetric Hebbian changes are observed. When evolving weights to a single pattern with unsigned states, the weights are not symmetric and not Hebbian – as you say. But I happen to know that in other conditions (e.g. when connections are costly) asymmetric interactions evolve even when the states are signed. So, question: Is there a reason for selection to create asymmetric weights in the heteroassociative task, does it require asymmetric weights (your Fig 1 suggests yes), and would asymmetric weights then evolve even in the signed state model? If we don’t know the answers to these questions, then Im not sure that all the rows of your Table 1 follow from the choice of signed/unsigned state models. As per 2 above, Im not sure that such questions/issues regarding the comparison of the modelling choices are the main contribution of this paper – in which case this doesn’t matter so much.

Statements regarding the symmetry is a straightforward property that follows from the analytical formulae and valid for the analytical models only. In case of signed states and Hebbian learning the formula is symmetric ($a\circ a$). The symmetry brakes by introducing {0,1} representation. The symmetry also breaks, irrespective of the representation, by adding heteroassociativity, cf. Eq. (3). We change the title of Table 1 to “*Comparison of the resulting analytic interaction matrices…*” to be more precise.

Q4. Since capacity and robustness are in the title….There is early (1980s) and recent work on Hop Net capacity (e.g. Folli, V., Leonetti, M., & Ruocco, G. (2017). “On the maximum storage capacity of the Hopfield model”. Frontiers in computational neuroscience, 10, 144. and refs therein)

We have inserted a new paragraph regarding to the problem of memory capacity and included the suggested references into the text. As our treatment is twofold (auto- and heteroassociativity) any direct comparison with previous results on the capacity of autoassociative systems is problematic.

Fig 6 top and middle supports your claims nicely wrt robustness under large perturbations. Im not sure that the bottom fig does though – If Im reading it right. It does have a larger tail in the limit of large perturbations, but the initial drop off is large – I don’t see how this supports the claim that neutrality increases robustness.

Indeed, the sentence (“*This is in sharp contrast to the standard Hebbian set-up, where there are no neutralities, due to the* $\{-1,+1\}$ *representation; see Table 1.*”) is not supported by the results; it has been removed from the Results section. We also include a short explanation on the difference between the robustness of evolutionary and analytical models against eliminating interactions. Thank you for pointing out!

Some other (optional) suggestions and thoughts for consideration are provided in the commented pdf

We incorporated most of your suggestions that improved the consistency and soundness of the manuscript.

In addition to the above changes, Fig 7 was corrected, because the analytic results in subfigures B and C were obtained with $\sigma=0.15$ instead of $\sigma=0.1$. These corrections did not affect the interpretation of the results.

In sum, this paper makes a very strong contribution to the topic, and very nicely written and easy to read - with strong technical analysis. I hope these suggestions above are helpful.

Thank you very much again!

Reviewer #2:

Review of the manuscript “Phenotypes to remember: Evolutionary developmental memory capacity and robustness”, by Szilágyi et al.

In the manuscript “Phenotypes to remember: Evolutionary developmental memory capacity and robustness”, the authors considered a gene-regulation network model that maps the initial patterns to given output patterns, in analogy with the neural network models, and then studied how evolution shapes such network.

I appreciate the authors’ motivation and relevance of the results to cell and developmental biology. Still, there are some issues to be revised, before the manuscript is considered as publication.

We really appreciate the thoughtful review of our manuscript. We found your comments and recommendations useful. We incorporated most of your suggestions into the manuscript. Please see our comments and answers below.

Q1: The Introduction presents a general grand view to connect the two celebrated studied by Turing. I understand the motivation well, but as compared with this grand view, but there exist extensive publication discussing such direction to bridge the gene-expression dynamical systems and neural-network type model, as both include a threshold-type dynamics. None of them are cited unfortunately, though. For instance,

Kauffman, S.A. J. Theor. Biol. 22 (1969) 437
Glass L. and Kauffman S. A., J. Theor. Biol., 39 (1973) 103.
Mjolsness E., Sharp D. H. and Reisnitz J., J. Theor. Biol., 152 (1991) 429.
Salazar-Ciudad I., Newman S. A. and Sole R. V., Evol. Dev., 3 (2001) 84.
Ciliberti S., Martin O.C., and Wagner A. (2007)PLOS Computational Biology, {\bf 3}, e15.
Kaneko K., PLoS ONE, 2 (2007) e434.

Indeed, there are related books by the above authors (Kauffman, Newman, Wagner, Kaneko), that include general discussion on dynamical-systems approach to development.

We modified the introduction and cited the relevant articles related to the dynamics of gene regulatory networks and associative neural networks and the intersection of these areas. Beside others we cited the articles you suggested.

Q2: Although, the authors discuss connection with, and possible difference from, neural networks (NN), somehow the related studies on NN are not mentioned. The first term in the matrix M in eq (3) or (4) is nothing but the Hopfield connection, \xi\xi (by suitably transforming [-1,1]to [0,1]; e.g., by setting \sigma=1/2 and setting \xi=a-1/2), whereas Hopfieild’s paper (Hopfield, J. J. (1982)PNAS 79, 2554-2558) is not cited, even in the Table. Of course, the connection of Hopfield model is symmetric, but there are several papers that extend the connection to make it asymmetric. (Indeed, the originally NN studies take the asymmetric connection).

We cited the classical Hopfield paper in connection to the autoassociative term of Eq. (3) right before Table 1.

* Indeed the asymmetric coupling form here is quite similar with that adopted by the study by Kurikawa T. and Kaneko K. (Europhysics Letters, 98(2012), 48002; PLoS Comp Biol. 9(2013) e1002943 ) , where NN in which the output pattern \xi is attracted under the input pattern \eta is designed (or learned) by setting the matrix (\xi+\eta)(\xi-\eta). Note that the matrix eq.4 by setting e-1/2=\eta a-1/2=\xi has the form (\xi+\eta)\xi. Hence, the difference between eq.(4) and Kurikawa’s model is just the absence of -\eta term. Indeed, in the latter model, the input \eta continues to be applied, whereas in the authors’ study it is applied only as an input, which leads to the absence of -\eta term.

Thank you drawing our attention to these relevant papers. We point out the connections right after Eq. (3) and inserted these into to references.

*On the other hand, if the connection is sparse, (or if there is no overlap in the input and output), the model could be considered as an extension of the Perceptron or hetero- associative memory model, where the connection term {\bf a}{\bf e} is generally adopted. Indeed, in the example the authors showed in the manuscript, there are no common components between e and a. I wonder if the model includes this non-overlapping as a constraint, or not.

We deliberately set the ***e*** and ***a*** vectors non-overlapping, to make the hetero-autoassociative task “harder”. For those gene that are expressed in both ***e*** and ***a*** the problem is biased toward an autoassociative task.

Q3: The memory capacity in NN has been extensively studied, whereas the capacity in the present model is not mentioned. As far as I guess from the result of N=50,100, 250, in Fig.3, the capacity in the example is about ~0.04N. It will be relevant to mention the capacity, as well as its dependence on the parameters, say sparsity \sigma.

According to your suggestion we have analyzed the memory capacity of our hetero/autoassociative system. We defined the capacity as the number of different developmental pathways (*Q*_{0.95}) can be learnt at an accuracy of *r*=0.95 Pearson correlation. We found that *Q*_{0.95} depends linearly on the system size (with *R*^2^>0.99), and the slope is the decreasing function of increasing $\sigma$. We inserted a new paragraph discussing our results and tangle the problems of comparison of our results with the standard results for Hebbian systems.

Q4: It is interesting that the evolution shapes the connection matrix similar to the analytically designed one. Still, it should be noted that the evolution of GRN has been investigated extensively (say, the last three papers in (1)), as well as in recent publications to evolve an input-output relationship in GRN.

We have now cited the following publications in the relevant parts of the Introduction and the Results sections.

Salazar-Ciudad I., Newman S. A. and Sole R. V., Evol. Dev., 3 (2001) 84.
Ciliberti S., Martin O.C., and Wagner A. (2007) PLOS Computational Biology, **3**, e15.
Kaneko K., PLoS ONE, 2 (2007) e434.

Q5: Although the authors mention the similarity between the evolved network and analytically designed one in Fig.4, the results in Fig.6 show clear difference between the two. Of course, as the two networks are different, the behavior could be different. Still, it is recommended to discuss possible origin of the discrepancy. For instance, the evolved network can include some additional connections that have neutral influence. If that is the origin of discrepancy, a possible procedure would be to extract the core part from the evolved network (i.e., remove paths, as long as the fitness is not decreased, as adopted in some earlier studies), and compare the behavior of the core network with that by the analytically designed one.

Note that as we mentioned in the text, Fig 5 panel B shows the average of 300 evolutionary runs, while the robustness analysis based on the resulting matrices of the individual evolutionary runs. The individual evolutionary realizations exhibit larger variance since there is no selection for on how the regulatory effects affecting a given gene are distributed amongst other genes which are similar regarding their expression in the embryo and adult states.

Consider an example with a single embryo-adult pair. All the genes can be divided into four distinct categories regarding their expression in the ***e*** and ***a*** states. The desired regulatory effects of the genes within these groups are the same. Regarding any affected gene, there is selection only on the sum, but not the distribution, of the regulatory effects of the genes within these groups. Therefore, in different evolutionary realizations the distributions are different, due to random drift.

Apparently the robustness is negatively affected by these differences as compared to the analytic matrix, especially in the case of nullifying (eliminating) matrix perturbations, see Fig. 7C. A less even distribution of regulatory effects implies smaller redundancy and compensation ability in turn against these types of perturbations.

Note that there was no selection incorporated into the present evolutionary model directly against these perturbations, therefore the evolutionary emergence of more robust matrices cannot be excluded in a more sophisticated model. However, due to the extensive scope of the issue of perturbations, and its computational requirements, such an analysis is beyond the scope of the present study.

We inserted a paragraph into the Results section regarding this issue.

Q6: Minor: In the caption of Fig.4, the authors refer to eq.(3). I wonder whether the analytic estimated is based on eq(4). Also some symbols K, \mu are not defined, if I have not looked over (K is a typos for N?).

*K* is the number of individuals in the population, while $\mu_{W}$ and $\mu_{e}$ are the variance of the mutation of the elements of the regulatory matrices and embryonic vectors, respectively. All three variables are introduced in the Evolutionary model subsection.

In addition to the above changes, Fig 7 was corrected, because the analytic results in subfigures B and C were obtained with $\sigma=0.15$ instead of $\sigma=0.1$. These corrections did not affect the interpretation of the results.
